# Supplementary material for: Hierarchical morphogenesis of swallowtail butterfly wing scale nanostructures
Source: eLife. 2023 Sep 28;12:RP89082. doi: 10.7554/eLife.89082 (PMC10538957; doi:10.7554/eLife.89082)
Supplement: Supplementary file 1. [file elife-89082-supp1.docx]

| Species | Sample no. | Sex | Treatment | Pupation stage when injected | Fate |
| --- | --- | --- | --- | --- | --- |
| *P. eurimedes* | 001 | F | - | - | Dead |
| *P. eurimedes* | 002 | M | 100uM CK-666 | 52% | Emerged* |
| *P. eurimedes* | 003 | M | DMSO control | 52% | Emerged* |
| *P. eurimedes* | 004 | M | 100uM CK-666 | 57% | Emerged* |
| *P. eurimedes* | 005 | M | 100uM CK-666 | 57% | Emerged* |
| *P. eurimedes* | 006 | M | DMSO control | 57% | Emerged* |
| *P. eurimedes* | 007 | M | 100uM CK-666 | 67% | Dead |
| *P. eurimedes* | 008 | M | 100uM CK-666 | 67% | Dead |
| *P. eurimedes* | 009 | M | DMSO control | 67% | Emerged* |
| *P. iphidamas* | 001 | M | 100uM CK-666 | 52% | Emerged* |
| *P. iphidamas* | 002 | F | 100uM CK-666 | 52% | Emerged* |
| *P. iphidamas* | 003 | M | DMSO control | 52% | Emerged* |
| *P. iphidamas* | 004 | M | 100uM CK-666 | 67% | Dead |
| *P. iphidamas* | 005 | M | 100uM CK-666 | 67% | Dead |
| *P. iphidamas* | 006 | M | DMSO control | 67% | Dead |
| *P. polytes* | 001 | M | 10uM CK-666 | 40% | Dissected |
| *P. polytes* | 002 | F | 10uM CK-666 | 40% | Dissected |
| *P. polytes* | 003 | F | 10uM CK-666 | 40% | Dissected |
| *P. polytes* | 004 | F | DMSO control | 40% | Emerged |
| *P. polytes* | 005 | M | 100uM CK-666 | 40% | Dissected |
| *P. polytes* | 006 | F | 100uM CK-666 | 40% | Dissected |
| *P. polytes* | 007 | F | 100uM CK-666 | 40% | Dissected |
| *P. polytes* | 008 | F | DMSO control | 40% | Dissected |
| *P. polytes* | 009 | F | 10uM CK-666 | 50% | Dissected |
| *P. polytes* | 010 | M | 10uM CK-666 | 50% | Dissected |
| *P. polytes* | 011 | F | 10uM CK-666 | 50% | Dissected |
| *P. polytes* | 012 | M | DMSO control | 50% | Emerged |
| *P. polytes* | 013 | M | 100uM CK-666 | 50% | Dissected |
| *P. polytes* | 014 | F | 100uM CK-666 | 50% | Dissected |
| *P. polytes* | 015 | F | 100uM CK-666 | 50% | Dissected |
| *P. polytes* | 016 | M | DMSO control | 50% | Dissected |
| *P. polytes* | 017 | M | 10uM CK-666 | 60% | Dissected |
| *P. polytes* | 018 | F | 10uM CK-666 | 60% | Dissected |
| *P. polytes* | 019 | M | 10uM CK-666 | 60% | Dissected |
| *P. polytes* | 020 | M | DMSO control | 60% | Dissected |
| *P. polytes* | 021 | F | 100uM CK-666 | 60% | Dissected |
| *P. polytes* | 022 | F | 100uM CK-666 | 60% | Dissected |
| *P. polytes* | 023 | M | 100uM CK-666 | 60% | Dissected |
| *P. polytes* | 024 | F | 100uM CK-666 | 60% | Dissected |
| *P. polytes* | 025 | M | DMSO control | 60% | Dissected |
| *P. polytes* | 026 | F | 10uM CK-666 | 70% | Dissected |
| *P. polytes* | 027 | F | 10uM CK-666 | 70% | Dissected |
| *P. polytes* | 028 | F | DMSO control | 70% | Dissected |
| *P. polytes* | 029 | F | 100uM CK-666 | 70% | Dissected |
| *P. polytes* | 030 | F | 100uM CK-666 | 70% | Emerged |
| *P. polytes* | 031 | F | - | - | Dead |
| *P. polytes* | 032 | F | 100uM CK-666 | 70% | Dissected |
| *P. polytes* | 033 | M | DMSO control | 70% | Dissected |
| *P. polytes* | 034 | M | 10uM CK-666 | 80% | Dissected |
| *P. polytes* | 035 | F | 10uM CK-666 | 80% | Dissected |
| *P. polytes* | 036 | F | DMSO control | 80% | Dissected |
| *P. polytes* | 037 | F | 100uM CK-666 | 80% | Dissected |
| *P. polytes* | 038 | F | 100uM CK-666 | 80% | Dissected |
| *P. polytes* | 039 | F | 100uM CK-666 | 80% | Dissected |
| *P. polytes* | 040 | F | DMSO control | 80% | Dissected |

*All *Parides* *eurimedes* and *P. iphidamas* were allowed to develop and emerge as adults and then dissected, unlike *Papilio polytes* pupae (see Methods).
